# Hand-held ultrasonography: An opportunity for “hands-on” teaching of medicine

**DOI:** 10.15694/mep.2018.0000103.1

**Published:** 2018-05-16

**Authors:** Victor Galusko, Owen Bodger, Emma Rees, Adrian Ionescu

**Affiliations:** 1 Swansea University Medical School; 2 College of Human and Health Sciences; 3 Morriston Cardiac Regional Centre

**Keywords:** Hand-held ultrasound, undergraduate medical education, echocardiography

## Abstract

This article was migrated. The article was marked as recommended.

**Background:** As ultrasound offers students an opportunity to study anatomy, physiology and pathophysiology actively, we used hand-held ultrasound (HHU) devices to augment current teaching of cardiac murmurs and pathology.

**Methods**: Three types of teaching sessions (of different duration) were explored: 1) compulsory teaching on cardiac murmurs (n=40); 2) extra-curricular teaching of cardiac murmurs (n=8); 3) extra-curricular ultrasound course (n=6). We assessed students’ ability to identify valvular lesions on auscultation, and anatomy and pathology on echocardiography, and sought qualitative feedback.

**Results:** Using echocardiography to teach murmurs improved murmur recognition by auscultation alone from 23% pre-test to 93% post-test (p=0.017). Students were able to identify major cardiac anatomical landmarks on echo images (57% vs 98% ( *p*=0.027) in the voluntary teaching session lasting 90 minutes, and 40% vs 82% ( *p*=0.027) after the 3 week cardiac ultrasound course. The mean accuracy for diagnosing cardiac pathology on a printed image alone after the 3 week ultrasound course was 71%. Students unanimously found the sessions useful and engaging, and reported they would like further teaching about using ultrasound.

**Conclusion:** Medical students found the sessions engaging, enjoyed this novel way of teaching and would like further teaching using ultrasound. Using hand-held ultrasound scanners to augment the teaching of cardiac murmurs to medical students is feasible and effective.

## Introduction

In the hands of a trained user, a hand-held ultrasound (HHU) device offers quick, accurate ^(1) (2)^ and cost-effective clinical assessment ^(3)^ by providing point-of-care ultrasound (POCUS) imaging ^(4) (5)^, which is often sufficient to change patient management ^(5) (6)^. As HHU use in clinical practice expands, doctors in training would benefit from early exposure to HHU scanning, and from acquisition of the skills to use HHU.

Many medical schools have already integrated ultrasound teaching into their curricula using high-end ultrasound devices ^(7) (8)^, and a “national ultrasound curriculum” has been developed in the USA ^(9)^. In the UK, the lack of allocated time in medical curricula and the absence of funding make such changes hard to implement ^(10) (11)^. HHU devices are cheaper, easier to operate, and are more portable than high-end, stationary ultrasound equipment, and therefore have the potential to assist the integration of ultrasound into medical school curricula.

The European Association of Echocardiography ^(12)^ supports the use of HHU in medical education; however, the literature available on the topic is limited. The published use of HHU devices has focused on teaching medical students to recognise a small number of mainly cardiac pathologies ^(13) (14) (15) (16)^. Few publications report the use of HHU to teach other organ areas ^(16) (17)^. Formal ultrasound imaging has been successfully used to improve the medical students’ knowledge of living anatomy and physiology ^(11) (18)^, as well as their motivation to learn ^(19)^. Whether the same is true for HHU, is not known.

Most of the published studies report the use of radiologists, cardiac sonographers and cardiologists to teach ultrasound to medical students. We set out to assess the feasibility of using a final-year medical student to teach the basics of cardiac ultrasound and to augment the teaching of cardiac murmurs using HHU, and we assessed the impact of HHU on student performance.

## Methods

### Structure of the teaching sessions

We conducted a systematic literature review on the use of HHU in medical education (in press) to inform the structure and objectives of the teaching sessions. We investigated the feasibility and views of medical students on the use of HHU to deliver cardiovascular teaching. To do this we conducted three teaching sessions of differing lengths - Table 1 summarises the three teaching sessions, their learning objectives and participant characteristics. The sessions varied in length, and therefore the depth of the subject cover. This was done to try and ascertain the depth of knowledge that needs to be delivered, and how best to deliver it (hands on vs didactic). Full details of the learning objectives are in Appendix 1-3 for the three respective teaching sessions.

### Using echocardiography to augment understanding of cardiac murmurs

#### Session 1

The session started with a test ( Appendix 4). Participants had to label landmarks of cardiac anatomy on printouts of frozen-frames of echocardiographic cross-sections, then they had to listen to various murmurs on a simulator (SimMan, Laerdal Medical Limited, Laerdal House, Orpington, UK) and formulate a diagnosis; finally, they were shown colour-flow mapping video loops illustrating the pathologies they had listened to and had the opportunity to change their diagnosis in the light of this supplementary information. After a teaching session lasting 1 ½ h and consisting of both didactic lectures and hands-on scanning, participants re-sat the pre-session test, using a different set of images and simulated murmurs.

### Using echocardiography to augment understanding of systolic cardiac murmurs

#### Session 2

The forty students attending the usual clinical skills teaching, were divided into groups of 6-10 students and rotated through the different clinical skills stations. One of these compulsory “stations” was a 25-minute, practical session teaching using the HHU and covering the pathophysiology of systolic murmurs. They gave qualitative feedback as well as free text comments upon completion of the session ( Appendix 5).

### Extracurricular HHU course

#### Session 3

Six students attended a total of five hours of teaching split over three weeks, one session per week. The aim was to familiarise students with echocardiography and its use in medical practice as well as to teach them to identify anatomy on printed echocardiography images, identify basic valvular pathology and severe LV systolic dysfunction. Students completed pre-test and a post-test questionnaires and gave qualitative feedback ( Appendix 6a- 6b). During the course we trialled projecting the images from the HHU using a high-definition web camera onto a larger screen to facilitate the teaching.

The ethic dimensions of the study were considered and discussed among the authors, and found to be similar to those applying to the standard teaching of clinical skills. This project was exempt from ethics approval at Swansea University because this was an evaluation of a teaching program approved by the Medical School and the principles of the Declaration of Helsinki were followed.

#### Facilitator

One of the authors (VG) who was, at the time, a final-year medical student devised and delivered the teaching sessions. VG received hands-on training in an echocardiography clinic supervised by a cardiologist, supplemented by self-directed learning (SDL) from books.

#### Assessment and feedback

The students provided feedback from the teaching sessions using a questionnaire with free text boxes and multiple choice questions. Appendix 4-6 contains the respective assessments and feedback questionnaires that the students received for the three teaching sessions. The ability of the students to identify cardiac anatomy and pathology on echocardiographic images was assessed in two of the teaching sessions - 1) the 90 minute cardiac murmur teaching session; 2) the 3-week cardiovascular ultrasound course. Only qualitative feedback and free text comments were collected for the 25-minute teaching session on systolic due to time constraints of the clinical skills teaching session. We did not assess students’ ability to recognise anatomy and physiology on echo, or their diagnostic ability to identify murmurs.

#### HHU scanner

The Vscan (GE Vingmed Ultrasound AS, Strandpromenaden 45, N-3191 Horten, Norway) uses a phased-array probe with a frequency range from 1.7 to 3.8 MHz; it weighs 390 g and fits inside a normal pocket. It displays grey-scale images with a fixed sector angle of 75°; the depth can be adjusted up to a 25 cm; colour flow has a fixed box size and a fixed pulse repetition frequency. There are no spectral Doppler capabilities.

#### Statistical analysis

Statistical analysis was performed using XLSTAT statistical package (Addinsoft, 28 West 27 ^th^ Street, New York, NY10001) to calculate the *p* values for the data (using Wilcoxon Signed Rank test where appropriate). The values were compared to the significance level of *p*<0.05.

## Results

### Using echocardiography to augment understanding of cardiac murmurs

#### Session 1

Eight students attended the session, and were taught as a group over an hour and a half. Prior to the teaching, students could correctly identify the anatomy of the heart in the apical 4-chamber (A4C) and parasternal-long axis (PLAX) views in 57% of cases (only one student had exposure to cardiology during a clinical attachment). Labelling of chambers correctly on echocardiography images improved from 57% before the teaching, to 98% (p=0.027) after. The average accuracy for identification of murmurs with auscultation alone improved from 29% before the teaching, to 93% afterwards (p=0.017). The availability of the echo images did not change the students accuracy of murmur detection during the assessment. All students enjoyed the teaching session, and the feedback showed that they found it relevant and useful (see Table 2 and 3, and Appendix 7).

### Using echocardiography to augment understanding of systolic murmurs

#### Session 2

During the clinical skills session we taught a total of forty students in rotations of 6-10 students at a time (25 minutes per group). They all found it “..useful to have hands-on murmur training” and the HHU allowed for an interactive teaching session with a “good mix of teaching approaches”. The live echo images enhanced the understanding of the pathophysiology of murmurs, and the students engaged well by asking questions on murmurs and clinical signs. Feedback from the session is summarised in Table 3. A selection of the comments from the session are given in Appendix 8. In summary, the students want more teaching about and with ultrasound, more hands-on time with the HHU, and more teaching on murmurs and how to pick them up on auscultation.

The time constraints of the session were evident, as many students did not get much “hands-on time” with the HHU device, and the large group size (6-10) did not aid the process. The session was too short for the number of students involved, as not all managed to get hands-on experience with the Vscan. We trialled projecting the HHU images onto a bigger screen using the high-definition web camera to facilitate the teaching, but were unsuccessful as the on-screen resolution was poor.

#### Extracurricular HHU course

As feedback from students clearly revealed an appetite for further instruction in cardiac ultrasound imaging, we designed and delivered a 3-week extra-curricular course on echocardiography. The course received overwhelmingly positive feedback ( Table 5 and Appendix 9) from the six student volunteers that attended it. Some of the students reported feeling more at ease with a student facilitator teaching a subject they were not familiar with.

Students developed the skills necessary to acquire and interpret the basic echocardiographic views of the heart (PLAX, PSAX and A4C) using HHU. Their ability to recognise the basic anatomy on an echo image of the heart improved significantly after teaching (40% vs 82% ( *p=*0.027)). Furthermore, their ability to recognise valvular pathology and severe LV systolic impairment improved from 4.2% to 71% ( *p*=0.027). The feedback and assessment results are summarised in Tables 5 and 6.

## Discussion

We report that using HHU to teach cardiac murmurs, alone or in addition to simulated cardiac sounds, is feasible and effective. Students were able to correctly recognise up to 93% of murmurs after the teaching, and were able to discern the cardiac anatomy on echocardiography images. Longer teaching sessions (lasting 5 hours) can teach students to recognise correctly identify cardiac pathology in most cases (71%). A trained medical student can act as an effective facilitator pitching the information at the right level, making those taught feel more at ease, and were more likely to ask questions. We received consistently good feedback from the students for both voluntary and compulsory (as part of clinical skills) teaching sessions. As doctors’ clinical skills decline ^(20)^, the HHU becomes a tool that can both enhance a physical examination ^(4)^ and improve the teaching of the physical examination and pathology.

### Group Size and Device Choice

Students found it hard to see the HHU screen when the groups were large (as large as 10 students), and not everyone had the chance to use the HHU in the available time. We trialled using a high-definition web camera to display images from the HHU on a bigger screen, as previously reported in order to stream images to an off-site expert ^(21)^, however we were unsuccessful as this achieved poor on-screen resolution. The 3-week teaching course was conducted using a single HHU device and accommodated 6 students well. A large amount of literature exists about the use of portable, as opposed to HHU, ultrasound in medical education ^(22) (23) (24) (25) (26) (27)^. Portable ultrasound equipment is available if larger group sizes need to be taught, some of which can be connected to a larger screen such as a projector ^(25)^.

### Limited availability of qualified trainers, and integrating the changes into the curriculum

Professionals such as cardiologists, radiologists and clinical scientists are probably best placed to teach ultrasound examination, however they are not readily available. The role of senior medical students and cardiac physiology/cardiac sonography students with prior experience in the area is potentially a fertile area of research ^(17) (28)^. As confirmed by the current study, medical students are able to acquire enough experience, and be knowledgeable enough to teach, at least to a basic level of competence.

To help and aid the learning process, a number of free online SDL resources are available, that have been tailored to medical student education, which include podcasts ^(17)^, i-books ^(29)^ and e-modules ^(30)^. SDL resources provide a valuable source of information to support learning ^(30)^. We suggest universities develop their own resources in the field which are tailored to their curriculum.

### Where do we go next with HHU?

**Figure 2.**
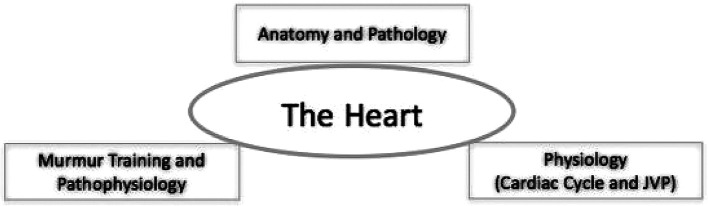
Important areas relating to the heart that we suggest should be taught using ultrasound in the curriculum

Most of literature published to date reports the use of HHU devices to teach medical students to recognise cardiac pathology ^(13) (14) (15) (16)^. Figures 2 and 3 illustrate that there are exciting concepts yet to be explored by educators when using ultrasound to teach medical students. The cardiac cycle can be brought to life by studying heart conditions with the aid of simulators and live scanning. Valvular dysfunction and septal defects can be studied using colour-flow mapping, and demonstrating its effects on the physiology of the heart. The jugular venous pulse (JVP) and its changes in patients with tricuspid regurgitation or congestive heart failure are probably relatively easy ‘targets’ for HHU-aided learning that need to be explored by future researchers.

We feel it is preferable to use ultrasound to complement clinical-skill teaching in medical school, rather than to teach the intricacies of ultrasound imaging. Ultrasound can enhance the current teaching by engaging multiple senses and allowing a more individualised self-paced learning experience. ^(31)^ This allows more effective teaching without necessitating for extra space in the medical school curriculum and would indirectly introduce the students to the basics of ultrasound. Furthermore, ultrasound can improve the students’ clinical knowledge by improving their knowledge of surface anatomy.

Teaching in the early years of medical school often focuses on surface anatomy, anatomy and physiology. Ultrasound can be used to link surface anatomy to the physical examination ^(7) (23)^ making the teaching clinically relevant, and complement pathology teaching in later years of medical school ^(7)^. Unique portability of HHU allows it to be used as a teaching tool both within the clinical lab setting and out in clinical practice.

There is still uncertainty about long-term retention of skills in ultrasound imaging ^(32)^ ^(33)^, and that may be a reason for which ultrasound teaching is still underdeveloped as its impact on whether it makes for more competent clinicians is unknown. However, as HHU is spreading widely in the medical community, threatening to render the stethoscope obsolete ^(34)^, it would be advantageous to become accustomed to this increasingly popular and powerful imaging modality early in a medical career.

## Limitations

We only delivered a single ‘cycle’ of HHU-aided teaching involving a limited number of students. The larger of the sessions was compulsory to all medical students whereas the other two were not, and therefore could have attracted only the interested students. Recurring usage over at least one year of study would be ideal before drawing firm conclusions. Ideally, assessing the impact of HHU on teaching medical students should incorporate formal results of tests and exams, or at least a broader array of competencies than we report here. Only then could we say with certainty that it is worthwhile to include HHU as a routine component of medical training

## Conclusion

The introduction of HHU as an aid to the teaching of cardiac murmurs markedly improved the accuracy of the clinical diagnosis of heart valve pathology. Students taking part in the teaching sessions found the teaching useful, relevant to their stage of training and understand the potential utility of HHU in their future clinical career. The resolution of the HHU devices appears sufficient for hands-on practice, to image major anatomical landmarks of the heart and to teach valvular pathology. The use of medical students, cardiac physiology students and junior doctors to teach ultrasound appears feasible but requires further research.

## Figures

**Figure 1.**
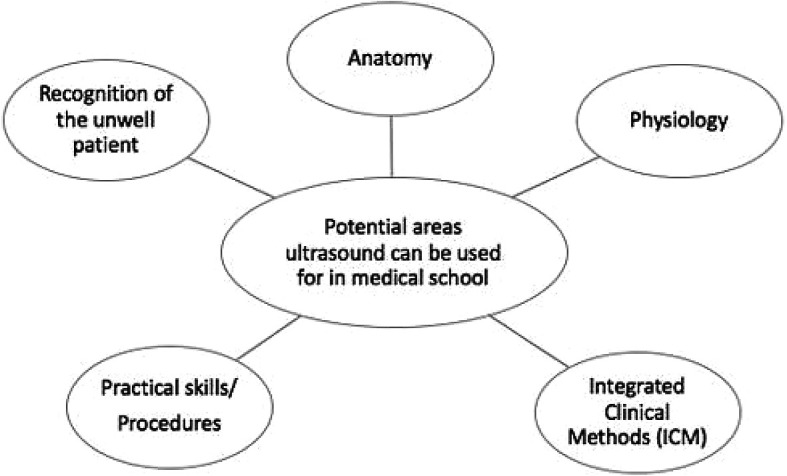
The figure below shows the five broad categories that ultrasound can be used for in medical education, a selection of these was trialled during our teaching sessions described in the report

## Tables

**Table 1.** Showing the content of the three sessions carried out. The first and third sessions were voluntary, whilst the second session was compulsory for all medical students attending clinical skills teaching. Didactic content consisted of a slide show based interactive teaching session, while hands-on sessions involved the students using Vscan to scan and explore the heart and its anatomy on normal subjects

	Using echocardiography to augment understanding of cardiac murmurs - Session 1	Using echocardiography to augment understanding of systolic murmurs - Session 2	Extracurricular HHU course - Session 3
**Main learning objectives**	To be able to identify the anatomy in PLAX and A4C views. To understand that valvular lesions can be visualised using HHU (focusing on AS, AR, MR and MS); and to link the pathophysiology of valvular lesions to understand the clinical signs they produce	To be able to identify the anatomy in PLAX and A4C views. To understand that valvular lesions can be visualised using HHU (focusing on AS and MR); and to link the pathophysiology of valvular lesions to understand the clinical signs they produce	To be able to identify the anatomy in PLAX, PSAX and A4C. To be able to identify valvular dysfuction (AS, AR, TR, MS, MR), to be able to assess LV function and be able to identify dilated LV
**Students**	2 ^nd^ year GEM students at the end of their pre-clinical years	1st year GEM students at the end of their pre-clinical year	GEM students at the end of their 2 ^nd^ pre-clinical year
**Facilitator**	Final year medical student
**Duration**	90 minutes	25 minutes	5 hours (delivered over 3 weeks)
**Percentage of didactic vs hands-on time**	45% - hands-on 55% - didactic	80% - hands-on 20% - didactic	50% - hands-on 50% - didactic SDL videos and handouts were freely available to supplement the teaching
**Setting**	Clinical lab scanning of volunteer students and use of simulation mannequin to produce murmur sounds	Clinical lab scanning of student volunteers	Clinical lab scanning of student volunteers
**Assessment**	The students labelled echo images in PLAX and A4C; and diagnosed murmurs by listening to four murmur sounds on a mannequin and then looking at an echo clip, marking which murmur they thought it was at each stage (pre- and post- intervention)	Quantitative feedback	Quantitative feedback + ability to interpret printed echo images and the anatomy visualised in PLAX and A4C. Diagnose three valvular lesions and a dilated LV on a printed image.

Key: PLAX - parasternal long axis; PSAX - parasternal short axis; A4C - apical 4-chamber; AS - aortic stenosis; AR - aortic regurgitation; MS - mitral stenosis; MR - mitral regurgitation; TR - tricuspid regurgitation; LV - left ventricle; GEM - graduate entry medicine; SDL - self-directed learning

**Table 2.** First teaching session improved the student’s ability to identify anatomy correctly on echocardiographic images and to detect valvular lesions by auscultation

	Average Student Score		
Average Student Score	Pre-test	Post-test	p-value
**Auscultation**	29%	93%	0.017
**Auscultation + Echo image**	29%	93%	0.017
**Chamber Identification**	57%	98%	0.027

**Table 3.** Qualitative feedback from the first teaching session

Feedback	
**Found the session useful**	100%
**I now feel more confident identifying murmurs**	100%
**I think HHU will influence my future practice as a clinician**	100%
**I want more teaching on ultrasound/with ultrasound**	100%
**I would like further teaching on:**	
**Jugular Venous Pulse**	75%
**Cardiac Cycle**	75%
**Cardiac Anatomy**	87.5%

**Table 4.** Qualitative feedback from the second teaching session

Feedback	
I found the session useful	100%
I feel I understand more about murmurs and the clinical signs they cause	98%
I feel I understand more about the pathophysiology of murmurs	100%
I feel more confident at interpreting murmurs	90%
I think the use of HHU devices will influence my future practice as a clinician	100%
I would like more teaching on ultrasound	98%

**Table 5.** Qualitative feedback from the third teaching session

Feedback	
I found the sessions interesting	100%
I found the sessions useful/relevant to my stage of training	100%
I understand more about the clinical indications for echocardiography, and how it’s used in clinical practice	100%
I see how HHU would be useful for me as a future clinician	100%
Improved my anatomy/physiology of the heart	100%
I would like more teaching on echocardiography	29%
The 3-week course was too long	0%

**Table 6.** After the third teaching session students’ scores increased significantly for correct identification of anatomy and detection of pathology in PLAX and A4C

	Mean student score		
	Pre-test	Post-test	*p*-value
**Identification of anatomy**	40.3%	81.9%	0.027
**Identification of pathology**	4.2%	70.8%	0.027

## Take Home Messages

• Medical students found the sessions engaging, enjoyed this novel way of teaching and would like further teaching using ultrasound.
• Using HHU scanners to augment the teaching of cardiac murmurs to medical students is feasible and effective.
• Final year medical students can be used as instructors.

## Notes On Contributors

Victor Galusko MBBCha, Owen Bodger PhDa, Emma Rees PhDb, Adrian Ionescu MDc

a - Swansea Medical School, Swansea University, Singleton Park, Swansea, UK

b - College of Human and Health Sciences, Swansea University, Swansea, UK

c - Morriston Cardiac Regional Centre, ABMU LHB, Swansea, UK

## Appendices

### Appendix 1. learning objectives for the first teaching session (using echocardiography to augment understanding of cardiac murmurs)

• To understand the uses of ultrasound in everyday practice
• To be aware the advantages and disadvantages of the HHU devices, and how these devices can be used to augment a standard physical examination and be used in emergency situations
• To understand the heart can be visualised in PLax and A4C, and the anatomy that can be seen
• To understand that valvular lesions can be visualised using ultrasound (focusing on AS, AR, MR and MS)
• To link the pathophysiology of valvular lesions to the findings on a standard physical examination

### Appendix 2. learning objectives for the second teaching session (using echocardiography to augment understanding of systolic murmurs)

• To understand the uses of ultrasound in everyday practice
• To be aware the advantages and disadvantages of the HHU devices, and how these devices can be used to augment a standard physical examination and be used in emergency situations
• To understand basic echocardiography views (PLax, PSax and A4C), and the anatomical structures visible
• To understand that valvular lesions can be visualised using ultrasound (focusing on the systolic murmurs (AS and MR))
• To link the pathophysiology of valvular lesions to the findings on physical examination

### Appendix 3. learning objectives for the extracurricular HHU 3-week course

#### Learning Objectives

**Basics (30-minute lecture) + Introduction to echocardiographic views (30-minute lecture)**

• Understand the importance of infection control and patient communication during ultrasound procedures
• Understand the importance of image archiving and documentation
• Become aware of the advantages and disadvantages of ultrasound, and recognize the limitations of portable ultrasound devices and when they should not be used
• Use surface anatomy to correlate probe placement with the cardiac structures visualized by echocardiography
• Be able to identify the structures in the heart, its chambers, vessels and pericardium on echocardiography images acquired in PLax, PSax and A4C
• Understand the indications for echocardiography and that formal echocardiography involves other views and is more accurate than HHU

**Echocardiography views and valvular anatomy (1 hour lecture + 1 hour hands-on practice)**

• Ability to identify the structures in the heart, its chambers, vessels and pericardium on given ultrasound images in PLAX, PSAX and A4C
• Ability to assess valvular function (stenotic and regurgitant lesions of the aortic, mitral, tricuspid valves)
• To understand that formal echocardiography is more accurate for the assessment of the severity of valvular lesions

**LV function (1 hour lecture + 1 hour hands-on practice)**

• To understand the basics of LV ejection fraction and the context in which it is important in clinical practice
• To be able to assess LV visually function and understand how this may affect patient management

### Appendix 7. feedback from the first teaching session

#### What was done well?

“Practical bit of teaching was great! Teaching was very clear and structured”

“Useful session because full teaching of murmurs was not covered in university well”

“Very useful - feel a lot more confident with murmurs now! Listening to SimMan was good + seeing echos”

“Murmur teaching - descriptions/characteristics of murmurs v. useful”

“Very clear description of murmurs - excellent teaching”

“Listening to the murmurs was really useful. Teaching was very effective and appropriate to our level of knowledge”

“Really helpful, did not know anything about echos and very little about ultrasound prior to this”

“Going over the heart sounds and effectively the pathophysiology of murmur was really good”

#### What can be improved on?

“May be too many murmurs to interpret took too long”

“Go a bit slower through the slides, may be a handout”

“I would have appreciated some sounds of the different murmurs being played as you discussed them”

“Teaching on the sound of each murmur was a bit fast, but still quite clear - might need a little longer to get my head around the sounds”

“Can the murmurs be played on loudspeaker before listening to them on the dummy - would be easier as we could get used to know what to listen for”

“The timing 3 weeks before exams”

### Appendix 8. some selected feedback from the second teaching session

“useful mnemonic to help to remember murmurs”

“well explained and practical”

“Useful to have a hands-on aspect to murmur training”

“Good mix of teaching approaches. Very interactive”

“taught in a nice simple way. Concise.”

“useful to have real-time images of the heart with explanation”

“It was useful to consider the anatomy of healthy hearts and compare them to diseased hearts”

“Clear and easy to understand, good structure”

“Very clear teacher. Subject knowledge was very good. Cool use of ultrasound”

“All of it was done well! Very knowledgeable guide!”

“Never seen the heart on echo before”

#### What can be improved on?

“perhaps make it a double station in the future”

“I need more practice at listening to murmurs”

“More on interpreting murmurs please”

“Some audio clips of murmurs would be useful!”

“Was good but would be helpful to be longer - still unsure on some murmurs”

“Large group today, so hard to see!”

“Would like more teaching to consolidate - maybe next year”

“would have liked some hands-on time”

“More time would be useful!”

“May be more time matching the anatomy to the scan”

“Slide show could be more in depth with sounds”

“Access to device for practice”

### Appendix 9. feedback from the third teaching session

#### General

“I have never done any ultrasound before so the course was an ideal introduction and a good recap of cardiac anatomy. Very clear teaching. VG made the topic interesting and aimed it at the right level”

“I was able to understand the views and what I was looking at. Practice was invaluable as was the exposure of seeing lots of scans

“It was useful to gain a basic understanding of ultrasound + how echocardiography is used, and I now know what I’m looking at generally. I also learnt a lot about cardiac diseases and why they result in the symptoms + structural alterations seen. Teaching was clear and easy to follow. Good to practice using the HHU!”

“Teaching + practical + cases helped with learning and understanding”

“More understanding of heart anatomy and good to have a go at echocardiography myself. Well taught. Victor has really good knowledge and good extra info for exams”

“Useful to understand the different views of the heart and what they can be used to measure/detect. The hands-on part was really good”

#### Improvements

“May be a big more hands-on time with the HHU”

“Perhaps arrangement for clinics where we could practice with the cardiac ultrasound technicians”

“A couple more machines might have been good. Then we could split up into smaller groups and get more hands-on time”

#### Peer instructor

“very good, knew about the level to pitch the teaching at”

“It didn’t feel pressured and I didn’t mind asking a stupid question”

“VG was great and his teaching was excellent. It made it applicable to our level of learning. Very interactive. He asks questions in a way that allow you to really think + understand the information”

“Really good - felt more comfortable taking guesses/ asking Qs. Whilst also can see he knows a lot about what he is teaching. Smaller group made the course comfortable/accessible”

“Really good. Think it helped as it was pitched at our level”

“Brilliant! Students tend to know the level to pitch information at as they have been in our position. I would like more peer teaching for other things (interpretation of blood results)”
