# Supplementary material for: Hand-held ultrasonography: An opportunity for “hands-on” teaching of medicine
Source: MedEdPublish (2016). 2018 May 16;7:103. [Version 1] doi: 10.15694/mep.2018.0000103.1 (PMC10699386; doi:10.15694/mep.2018.0000103.1)
Supplement: Peer review history

# Reviewer report: Reviewer response for version 1

**DOI:** 10.21956/mep.19566.r29023

**Published:** 2019-03-09

**Authors:** Trevor Gibbs

**Affiliations:** 1 AMEE

This review has been migrated. The reviewer awarded 4 stars out of 5 I quite enjoyed reading this paper and its purpose was very worthy. However I did find it difficult to read because I felt that the authors had over-stretched themselves by making their research too complicated. Although I can easily see that the use of HHU added to the understanding of cardiac anatomy and clinical diagnoses, I was at a loss to see which element of the teaching package produced the final conclusion. I feel that, like one of my co-reviewers, that the research could have been made much simpler by the use of a control group and comparing specific elements of the teaching activity. However I do feel that it is a worthy paper to read despite these limitations.

Reviewer Expertise:

NA

No decision status is available

# Reviewer report: Reviewer response for version 1

**DOI:** 10.21956/mep.19566.r29021

**Published:** 2018-10-10

**Authors:** Cristina Cernei

**Affiliations:** 1 Oxford Deanery

This review has been migrated. The reviewer awarded 5 stars out of 5 A very practical and ingenious idea to better medical education and improve clinical practice for junior doctors. It would be great if this could be implemented everywhere in the future. I highly recommend this article.

Reviewer Expertise:

NA

No decision status is available

# Reviewer report: Reviewer response for version 1

**DOI:** 10.21956/mep.19566.r29022

**Published:** 2018-06-08

**Authors:** David Bruce

**Affiliations:** 1 None

This review has been migrated. The reviewer awarded 3 stars out of 5 The authors set out to measure improvements in student learning and detection of cardiac murmurs using hand held ultra-sound (HHU) as a teaching aid. This is a thoughtful plan and a paucity of literature on this subject indicates the need for such a study. However I think the study design is over ambitious. The different cohorts of students participating in each session and variation in the amount teaching received and the teaching methods used made it difficult as a reader to draw conclusions. As each teaching session included more than the HHU teaching aid, the improvements in post test scores are a measure of student learning from the whole experience. And while overall we see student satisfaction with HHU teaching and evidence of learning which is very positive, if the purpose of the study is to demonstrate the added value of an intervention (HHU as a teaching aid), the inclusion of a control group would have strengthened this paper.

Reviewer Expertise:

NA

No decision status is available
